# Supplementary material for: Quadrupole anomalous Hall effect in magnetically induced electron nematic state
Source: Nat Commun. 2023 Dec 8;14:8074. doi: 10.1038/s41467-023-43543-1 (PMC10709597; doi:10.1038/s41467-023-43543-1)
Supplement: Supplementary file 1 — Supplemental Information [file 41467_2023_43543_MOESM1_ESM.pdf]

Supplemental Information for "Quadrupole anomalous Hall effect in magnetically induced electron nematic state"

Hiroki Koizumi,[1,2,3] Yuichi Yamasaki,[4] and Hideto Yanagihara[1,5]

[1]*Department of Applied Physics, University of Tsukuba, Tsukuba, Ibaraki 305-8573, Japan*

[2]*Research Center for Magnetic and Spintronic Materials (CMSM), National Institute for Materials Science (NIMS), Tsukuba, Ibaraki, 305-0047, Japan*

[3]*Center for Science and Innovation in Spintronics (CSIS), Tohoku University, Sendai 980-8577, Japan*

[4]*Research and Services Division of Materials Data and Integrated System (MaDIS), National Institute for Materials Science (NIMS), Tsukuba, Ibaraki, 305-0047, Japan*

[5]*Tsukuba Research Center for Energy Materials Science (TREMS), University of Tsukuba, Tsukuba, Ibaraki 305-8573, Japan*

Supplementary Note 1. SAMPLE CHARACTERISATION

We confirmed the composition ratio of the fabricated $NiCo_2O_4$ thin film using inductively coupled plasma mass spectrometry. The composition ratio exhibiting an anisotropic transverse resistivity (TR) was clarified as Ni:Co= 1.035 : 1.965, whereas that of the sputtering target was 1 : 2. The result suggests that the sputtering yield of Ni is slightly richer than that of Co during reactive sputtering.

The film structure was characterised using reflection high-energy electron diffraction and X-ray diffraction. Supplementary Figure 7**a** shows the θ-2θ X-ray diffraction scan using Co $K_{\alpha 1}$ (λ = 0.1789 nm) around $MgAl_2O_4$ and $NiCo_2O_4$ 004 Bragg reflections. The film thickness, estimated from the X-ray reflectivity, is 50 nm. The *a*- and *c*-axis lattice constants are 8.08 and 8.20 Å, respectively. The reciprocal space mapping contour plot of x-ray diffraction intensity, in Supplementary Fig. 7**b** reveals consistent in-plane lattice constant of the thin film with that of the substrate. Therefore, the thin film is confirmed to be coherently grown on the substrate. Supplementary Fig. 7**f** shows atomic force microscopy (AFM) images. A smooth surface morphology is confirmed and the typical root mean square roughness and peak-to-valley distance are 0.3 Åand 3.6 Å, respectively.

Sample dependence was found for the QuadAHE in $NiCo_2O_4$ thin films. The magnitude and magnetic field dependence of QuadAHE were sample dependent, but QuadAHE themselves could be observed in all samples that form conical magnetic structures. To check the influence of substrate quality, we fabricated some samples using substrates obtained from different providers. Although their AHE showed a different curve shape, the reproducibility of QuadAHE itself was confirmed. The data in this paper represent results obtained from two of those samples; Supplementary Fig. 6 shows the data obtained from one sample; the others are from another sample showing the largest QuadAHE within the fabricated samples.

Supplementary Note 2. ELECTRICAL CONDUCTIVITY CHARACTERISTICS

The mixed-valence of cations gives them relatively high electrical conductivity [1]. Supplementary Figure 7**c** shows the temperature dependence of longitudinal conductivity σ_L at zero magnetic fields with $\mathbf{J} \parallel [010]$. The conductivity is approximately half of that measured

in previous studies [2, 3]. Since the resistivity variation upon the application of a magnetic field is much smaller than that upon temperature changes, the anomaly is not directly related to the transition from the Néel-type collinear to the Yafet-Kittel-type non-collinear spin reorientation [1, 4]. Supplementary Figure **7d** shows the temperature dependence of AHE conductivity (σ_{AHE}), appearing below the Curie temperature of 310 K. In the present thin film, σ_{AHE} monotonically decreases below $T_S \approx 130$ K due to the magnetic structure changes from the Néel- to the Yafet-Kittel-type state. However, Kan *et al.* reported that σ_{AHE} is almost constant at low temperature [3], whereas Chen *et al.* reported that the sign of σ_{AHE} can be reversed by lowering the temperature in thinner $NiCo_2O_4$ films [2, 5]. Previous reports have been of stoichiometric $NiCo_2O_4$ thin film samples exhibiting perpendicular magnetic anisotropy, whereas the present sample is an off-stoichiometric film. Therefore, the difference in σ_{AHE} is assumed to be correlated with the ratio of antisite Ni^{3+} distribution.

Supplementary Note 3. MAGNETORESISTIVITY ANALYSIS

The observed magnetoresistance (MR) for the applied magnetic field $\mathbf{H} = (0, 0, H)$ is denoted as $\rho^{\pm}_{ij}(H)$ with applied current direction j, detected electric field direction i with $i, j = x : [100]$, $y : [010]$, $x' : [110]$, $y' : [\bar{1}10]$, and positive (+) or negative (−) magnetic field applied during cooling (H_{FC}). By antisymmetrisation/symmetrisation analyses, we obtain the antisymmetric- and symmetric MR as

$$\rho^{\mathrm{O}\pm}_{ij}(H) \equiv \frac{1}{2}\left\{\rho^{\pm}_{ij}(H) - \rho^{\pm}_{ij}(-H)\right\}, \tag{1}$$

and

$$\rho^{\mathrm{E}\pm}_{ij}(H) \equiv \frac{1}{2}\left\{\rho^{\pm}_{ij}(H) + \rho^{\pm}_{ij}(-H)\right\}, \tag{2}$$

respectively. The symmetric MRs do not depend on H_{FC}; hence, $\rho^{\mathrm{E}+}_{ij}(H) = \rho^{\mathrm{E}-}_{ij}(H)[\equiv \rho^{\mathrm{E}}_{ij}(H)]$. The observed antisymmetric TRs are decomposed into anisotropic (OA) and isotropic (OI) parts as $\rho^{\mathrm{O}\pm}_{ij} = \rho^{\mathrm{OI}\pm}_{ij} + \rho^{\mathrm{OA}\pm}_{ij}$ with

$$\rho^{\mathrm{OA}\pm}_{ij}(H) \equiv \frac{1}{2}\left\{\rho^{\mathrm{O}\pm}_{ij}(H) - (-\rho^{\mathrm{O}\pm}_{ji}(H))\right\}, \tag{3}$$

and

$$\rho^{\mathrm{OI}\pm}_{ij}(H) \equiv \frac{1}{2}\left\{\rho^{\mathrm{O}\pm}_{ij}(H) + (-\rho^{\mathrm{O}\pm}_{ji}(H))\right\}, \tag{4}$$

respectively. From the results in this study, we estimate that $\rho_{x'y'}^{\mathrm{OA}\pm}(H) = 0$, $\rho_{xy}^{\mathrm{OA}+}(H) = -\rho_{xy}^{\mathrm{OA}-}(H)$, and $\rho_{xy}^{\mathrm{OI}\pm}(H) = \rho_{x'y'}^{\mathrm{OI}\pm}(H)$. As shown in Supplementary Figure 8**a**, the behaviour of $\rho_{x'y'}^{\mathrm{OI}\pm}(H)$ is consistent with magnetization hysteresis loop one measured by the vibrating-sample magnetometry (VSM) at 10 K. The difference observed by reversing H_{FC} is expressed as

$$\rho_{ij}^{\mathrm{OA}\Delta}(H) \equiv \frac{1}{2}\{\rho_{ij}^{\mathrm{O}+}(H) - \rho_{ij}^{\mathrm{O}-}(H)\}, \tag{5}$$

suggesting $\rho_{xy}^{\mathrm{OA}\Delta}(H) = \rho_{[xy]}^{\mathrm{OA}+}(H)$ [see Supplementary Fig. 3] and $\rho_{x'y'}^{\mathrm{OA}\Delta}(H) = 0$.

In general, it is impossible to totally remove experimental errors, such as misalignment of Hall bar and film inhomogeneity, from the symmetric TR $\rho_{ij}^{\mathrm{E}}(H)$. However, it appears only below temperature T_S, concurrent with the magnetic transformation, as shown in Fig. 3**d** and Supplementary Fig. 4. Moreover, the sign shows the antisymmetric response with respect to H_{FC}, as shown in Supplementary Fig. 6, suggesting that the influence of these artefacts on the symmetric TR is negligibly small.

Supplementary Note 4. MAGNETIC FIELD COOLING PROCEDURE

The QuadAHE is sensitive to the sign, angle, amplitude, and initial temperature of the magnetic field H_{FC} applied during cooling. Initially, we tuned the angle between the applied magnetic field and the direction normal to the sample plane by measuring the angle dependence of transverse voltage at 300 K using the rotator probe with one variable axis around the [110] direction. The other axis of rotation around the [$\bar{1}$10] axis was not present; therefore, the magnetic fields were not adjusted to be perfectly oriented in the perpendicular direction.

The tilted magnetic field due to the misalignment of the measurement device contributes to a single domain in $\tilde{T}_u$ MTQ conical magnet. This can be confirmed by comparing the QuadAHE with that measured after rotating 90° within the plane without changing the wiring of the 4-terminal electrodes used for measuring resistivities, as shown in Supplementary Fig. 6. In both setups, the electric current is applied for $\mathbf{J}||[100]$ and cooling field is $\mu_0 H_{\mathrm{FC}} = \pm 9$ T. The QuadAHE and symmetric TR show sign reversal only by rotating the sample, even with identical magnetic field cooling procedures. It is presumed that the tilted magnetic field can uniquely select the magnetic structure of the MTQ conical, and its sign is determined by whether the in-plane component is the [110] or [$\bar{1}$10] direction. Such

responses can be understood by Dzyaloshinskii-Moriya interactions, as discussed below.

The influence of the $\mu_0 H_{\rm FC}$ magnitude at 300 K on the QuadAHE is shown in Supplementary Fig. 8**b**. We performed the cooling procedure with different $\mu_0 H_{\rm FC}$ = 0, 0.1 and 9 T. AHE curves with $\mu_0 H_{\rm FC}$ = 0.1 T and 9 T are consistent, while that with $\mu_0 H_{\rm FC}$ = 0 T exhibits a different behaviour. Even with cooling field as low as 0.1 T, it is important to homogenise the MTQ conical domain, and once cooled, the single domain state remains robust during AHE vs. H measurements with applied higher magnetic field up to 9 T.

Next, we investigate the critical temperature and cooling field to switch the MTQ domain. The influence of the initial field cooling temperature on the QuadAHE is shown in Supplementary Fig. 8**c**. In the initial process, the sample was cooled from 300 K to 5 K with $\mu_0 H_{\rm FC}$ = +0.1 T, and the magnetic field dependence of AHE was measured. Next, the sample was warmed to selected temperatures (150 and 175 K) under zero field with no temperature overshoot and then cooled to 5 K with the opposite field of $\mu_0 H_{\rm FC} = -0.1$ T; the magnetic field dependence was again measured at 5 K. The QuadAHE is completely reversed when the temperature is raised up to 175 K but it is only partially reversed at up to 150 K, indicating that the initial state is partially maintained. The QuadAHE signal mostly disappears at $T_S \approx 130$ K, but the single domain feature of $\tilde{T}_u$ MTQ conical magnet survives up to 150 K.

Supplementary Figure 8**d** shows the QuadAHE dependency on the $H_{\rm FC}$ magnitude. The initial status was the same as described above. Afterward, the sample was warmed up to 150 K in the zero field with no overshoot and cooled to 5 K with $\mu_0 H_{\rm FC} = -0.1$ and -1 T, and TR was measured at 5 K. The QuadAHE was partly and completely reversed with -0.1 T and -1 T, respectively. Hence, warming up to 150 K and applying a magnetic field of at least $|\mu_0 H_{\rm FC}| = 1$ T will reverse the sign of MTQ $\tilde{t}_u$.

Supplementary Note 5. MAGNETIC STRUCTURE

While the anisotropic Hall effect can be attributed to the B-site pyrochlore lattice due to the $\mathcal{C}_4\mathcal{T}$ symmetry, the symmetric TR is attributed to the A-site diamond lattice. The emergence of the symmetric TR below T_S is interpreted as a change from the Néel-type magnetic structure to the Yafet-Kittel-type two-dimensional non-collinear magnetic structure [6] owing to changes in magnetic anisotropy [4] (Fig. 3**d**). Spin canting of A-site along

the [110] or [$\bar{1}$10] direction would remove tetragonal $\mathcal{C}_4$ symmetry, resulting in anisotropic TR. Since the sign of symmetric TR depends on the direction of sample mount and not on $H_{\rm FC}$ [Supplementary Fig. 6], the canting direction of A-site would be determined by the tilted $H_{\rm FC}$ direction. $NiCo_2O_4$ shows the ferrimagnetic order with the A- and B- site spins pointing in opposite directions. The antiferromagnetic coupling between the two sites J_{AB} acts as an internal magnetic field against $\mathbf{H}$ on the B-site [7, 8]. Below T_Q, the A-site cant angle widens, and the internal magnetic field decreases, allowing the non-collinear magnetic structure of the B-site owing to the DMI [Fig. 3**d**]. A magnetic field appears to widen the cant angle of the B-site; however, the opposite internal magnetic field, due to the decrease in the A-site cant angle, is increasingly affected, suppressing the B-site cant angle. Such a magnetic field response explains why $\rho_{ij}^{\rm OA}$ is observed at lower magnetic fields than $\rho_{ij}^{\rm E}$.

Supplementary Note 6. ANISOTROPIC RESISTIVITY BY MAGNETIC TOROIDAL QUADRUPOLE

We consider a minimal model based on spin splitting induced by the magnetic toroidal quadrupole (MTQ) order with $4'/mm'm$ symmetry. A minimal model of the Hamiltonian is expressed as [9],

$$\mathcal{H} = \left[\frac{\hbar^2}{2m}\left\{\mathbf{k}^2\sigma_0 + t_{xy}(k_x^2 - k_y^2)\sigma_z\right\} + m_z\sigma_z\right] c^\dagger_{\mathbf{k}\sigma}c_{\mathbf{k}\sigma} \tag{6}$$

with the Pauli matrixes σ_0, σ_x, σ_y, and σ_z. t_{xy} and m_z indicate the T_{xy} MTQ and M_z MD order parameters, respectively. Here, we consider only $k_z = 0$ for simplicity, with no spin mixing. Since the experimental results can be attributed to the $\tilde{T}_u$ $(= T_u + M_{xyz})$ MPG with $4'/mmm'$ symmetry, we consider the coordinate system of Eq. (6) rotated by 45° [see Supplementary Fig. 5]. Then, the Hamiltonian can be rewritten as Eq. (3) in the main text, and the energy dispersion is calculated as

$$\mathcal{E}_{\mathbf{k}\pm} = \frac{\hbar^2}{2m}\left(\mathbf{k}^2 \pm 2\tilde{t}_u k_x k_y\right) \pm m_z \tag{7}$$

where $\tilde{t}_u$ is the MTQ order parameter of $4'/mmm'$ and the sign + (−) indicates up (down) spin bands. The spin band is split by the MTQ order, and the Fermi surface is elliptically distorted where the semi-major axis is rotated by 90° between up and down spin bands, as shown in Fig. 3**f**.

Next, we consider the transport properties of magnetic Bloch electrons by combining the semiclassical and Boltzmann equations with a constant relaxation time ($\tau \equiv \tau_{\mathbf{k}}$) approximation,

$$\dot{\mathbf{k}} \cdot \frac{\partial f_{\mathbf{k}}}{\partial \mathbf{k}} = -\frac{f_{\mathbf{k}} - f_0}{\tau} \tag{8}$$

with a distribution function $f_{\mathbf{k}}$. The semiclassical equations of wave packets with a uniform electric field ($\mathbf{E}$) and without an external magnetic field are described by [10, 11]

$$\dot{\mathbf{r}} = \frac{1}{\hbar}\frac{\partial \mathcal{E}_{\mathbf{k}}}{\partial \mathbf{k}} - \dot{\mathbf{k}} \times \mathbf{\Omega}_{\mathbf{k}} \tag{9}$$

$$\dot{\mathbf{k}} = -\frac{e}{\hbar}\boldsymbol{E}, \tag{10}$$

where $\mathcal{E}_{\boldsymbol{k}}$ and $\mathbf{\Omega}_{\mathbf{k}}$ are the energy dispersion and Berry curvature, respectively. The linear response in the electric field is obtained as $\mathbf{j} = \mathbf{j}^D + \mathbf{j}^\Omega$ with

$$\mathbf{j}^D = \frac{e^2\tau}{\hbar^2}\int_{\mathbf{k}} \partial_{\mathbf{k}}\mathcal{E}_{\mathbf{k}} \left(\boldsymbol{E}\cdot\partial_{\mathbf{k}}\mathcal{E}_{\mathbf{k}}\right)\left(-\frac{\partial f_0}{\partial \mathcal{E}_{\mathbf{k}}}\right), \tag{11}$$

$$\mathbf{j}^\Omega = -\frac{e^2}{\hbar}\int_{\mathbf{k}} f_0\left(\mathbf{E}\times\mathbf{\Omega}_{\mathbf{k}}\right), \tag{12}$$

and $\int_{\mathbf{k}} \equiv \int d^k k/(2\pi)^d$. The first equation is the semiclassical contribution to the conductance expressed by the electronic group velocities. In a low-symmetric electronic structure, it can provide a longitudinal current and a finite contribution to the transverse current as discussed below. The second equation expresses the Berry curvature monopole contribution to the intrinsic anomalous Hall effect [11].

From Eqs. (7) and (9), a charge current owing to the applied electric field $\mathbf{E} = E_0(\cos\varphi,\ \sin\varphi)$ in the $\tilde{T}_u$ MTQ order is calculated as

$$\begin{pmatrix} j_{c||} \\ j_{c\perp} \end{pmatrix} = \sigma_D E_0 \begin{pmatrix} 1 + \tilde{t}_u^2 + 2\Delta n \tilde{t}_u \sin 2\varphi \\ 2\tilde{t}_u \Delta n \cos 2\varphi \end{pmatrix} \tag{13}$$

and a spin current as

$$\begin{pmatrix} j_{s||} \\ j_{s\perp} \end{pmatrix} = \sigma_D E_0 \begin{pmatrix} \Delta n(1 + \tilde{t}_u^2) + 2\tilde{t}_u \sin 2\varphi \\ 2\tilde{t}_u \cos 2\varphi \end{pmatrix}, \tag{14}$$

with $\sigma_D = \bar{n}e^2\tau/m$. Here, $\bar{n} = n_\uparrow + n_\downarrow$ and $\Delta n = (n_\uparrow - n_\downarrow)/(n_\uparrow + n_\downarrow)$, with $n_\uparrow$ ($n_\downarrow$) being the number of electrons in the up (down) spin bands. The results correspond to the charge and spin conductivity tensors listed in Supplementary Table I. Using Eq. (13) and assuming

$|\sigma_\perp| \ll |\sigma_\parallel|$ ($|\tilde{t}_u| \ll 1$), the φ-dependent resistivity tensor ρ can be derived from the inverse conductivity tensor σ as

$$\rho(\varphi) \approx -\frac{2\tilde{t}_u \Delta n}{\sigma_D^2} \begin{pmatrix} \sin 2\varphi & \cos 2\varphi \\ \cos 2\varphi & -\sin 2\varphi \end{pmatrix} \tag{15}$$

Therefore, the anisotropic resistivity is obtained as Eq. (5) in the main text.

Supplementary Note 7. DZYALOSHINSKII-MORIYA INTERACTION IN PYROCHLORE LATTICE

Since the midpoint of the two nearest-neighbour B-site cations is not the centre of inversion symmetry in the pyrochlore structure, there is a nonzero DM vector $\mathbf{D}_{ij}$ between the i- and j-th sites. Based on Moriya's rule, the $\mathbf{D}_{ij}$ vector is perpendicular to the displacement vector $\mathbf{r}_{ij} = \mathbf{r}_j - \mathbf{r}_i$ and within the mirror plane if it exists between neighbouring cations [12]. The sign is determined by the potential gradient around the bonding cations and spin-orbit interaction. Supplementary Figure 9**a** shows the $\mathbf{D}_{ij}$ vectors on a single tetrahedron of the pyrochlore lattice [13–15]. The difference in the in-plane and out-of-plane lattice constant due to epitaxial distortion results in different DM vectors magnitudes, $D_\parallel \neq D_\perp$ with $D_\parallel = |\mathbf{D}_{12}| = |\mathbf{D}_{14}| = |\mathbf{D}_{23}| = |\mathbf{D}_{34}|$ and $D_\perp = |\mathbf{D}_{13}| = |\mathbf{D}_{24}|$. Supplementary Figures 9**b** and 9**c** depict the DMI energy on each B-B bond in the four conical magnetic structures with a conical angle of $\theta_c = 30°$ for $M_z > 0$ and < 0, respectively. The sign and magnitude of DMI energy

$$E_{\mathrm{DMI}} = \sum_{i<j} \mathbf{D}_{ij} \cdot (\mathbf{S}_i \times \mathbf{S}_j) \tag{16}$$

is indicated by the colour (red or blue) and radius. The DMI energy variations in the four magnetic states $(\mathrm{sgn} m_z, \mathrm{sgn} \tilde{t}_u) = (+1, +1), (+1, -1), (-1, +1)$ and $(-1, -1)$ are shown in Fig. 4**c**. In contrast, Supplementary Fig. 9 indicates the DMI energy variation between $(\mathrm{sgn} m_z, \mathrm{sgn} \tilde{T}_X) = (+1, +1)$ and $(-1, +1)$ states for t_X, which are the order parameters of $\tilde{T}_u$, T_{xy}, M_z^α and T_v. The DMI energy on each bond is isotropic within the (001) plane for M_z^α and T_v conical magnet, whereas anisotropic for $\tilde{T}_u$ and T_{xy}.

Supplementary Note 8. ENERGY OF TILTED MTQ CONICAL STRUCTURE

Unit vectors of the magnetic moment for the $\tilde{T}_u$ MTQ conical magnetic structure on the B-site pyrochlore lattice are parameterised by the conical angle θ_c and a phase angle φ_c as follow,

$$\hat{\mathbf{S}}_1 = (\cos\varphi_c \sin\theta_c, \sin\varphi_c \sin\theta_c, \cos\theta_c) \tag{17}$$

$$\hat{\mathbf{S}}_2 = (-\sin\varphi_c \sin\theta_c, \cos\varphi_c \sin\theta_c, \cos\theta_c) \tag{18}$$

$$\hat{\mathbf{S}}_3 = (-\cos\varphi_c \sin\theta_c, -\sin\varphi_c \sin\theta_c, \cos\theta_c) \tag{19}$$

$$\hat{\mathbf{S}}_4 = (\sin\varphi_c \sin\theta_c, -\cos\varphi_c \sin\theta_c, \cos\theta_c), \tag{20}$$

with $\varphi_c = n\pi + \pi/4$ (n=integer). The total magnetic energy $E(\theta_c, \varphi_c)$, which includes the isotropic exchange interaction $J_{bb}\mathbf{S}_i \cdot \mathbf{S_j}$, the single-ion anisotropy, and the DM interaction, is independent of φ_c. Here, we consider the conical magnet tilted by a small angle θ_t around the vector $\hat{\mathbf{n}} = (-\sin\varphi_t, \cos\varphi_t, 0)$ with keeping the conical angle θ_c. Such tilting results in changes in the total magnetic energy, and the energy difference between the MTQ conical and that with opposite $\tilde{T}_u$ sign, *i.e.* $\Delta E \equiv E(\theta_c, \varphi_c) - E(\theta_c, \varphi_c + \pi)$, is derived as

$$\Delta E = (-1)^n \delta D \theta_t^2 \sin 2\theta_c \sin 2\varphi_t \tag{21}$$

with $\delta D = D_{\parallel} - D_{\perp}$. The result suggests that the stable sign of MTQ is determined not by the sign of θ_t but by the sign of M_z and whether the conical axis tilts toward [110] or [$\bar{1}$10] direction. The energy difference is the origin of $\tilde{T}_u$ MTQ conical single domain formation due to the tilted H_{FC}.

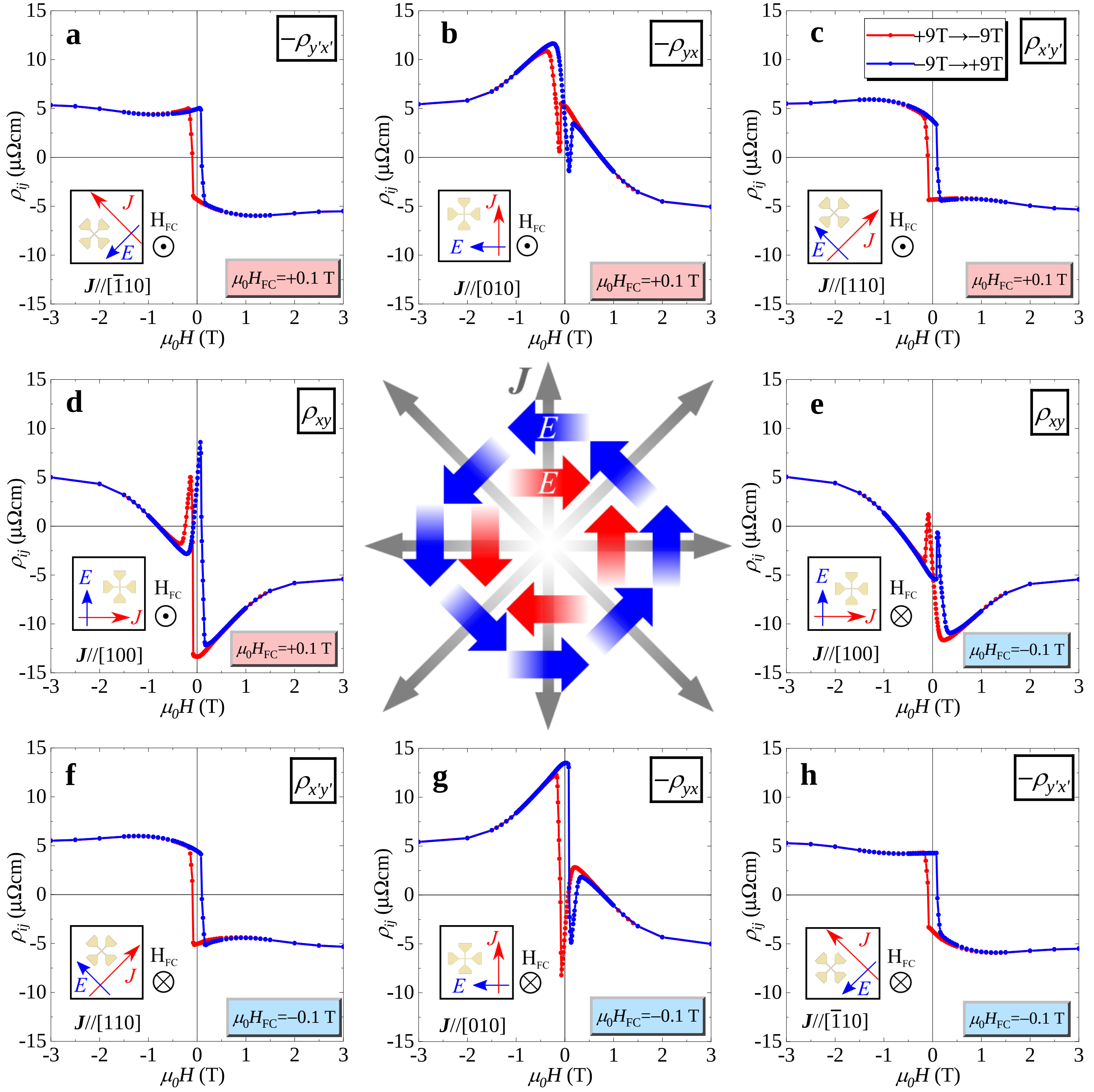

Supplementary Fig. 1. Magnetic field dependence of transverse resistivity (TR) ρ_{ij} at 5 K with changing current direction and cooling field. Currents are applied parallel to (**a**, **h**) **J**$||[\bar{1}10]$, (**b**, **g**) **J**$||[010]$, (**c**, **f**) **J**$||[110]$, and (**d**, **e**) **J**$||[100]$ axes. TR was measured after field cooling at (**a-d**) $\mu_0 H_{\mathrm{FC}} = +0.1$ T and (**e-h**) $\mu_0 H_{\mathrm{FC}} = -0.1$ T.

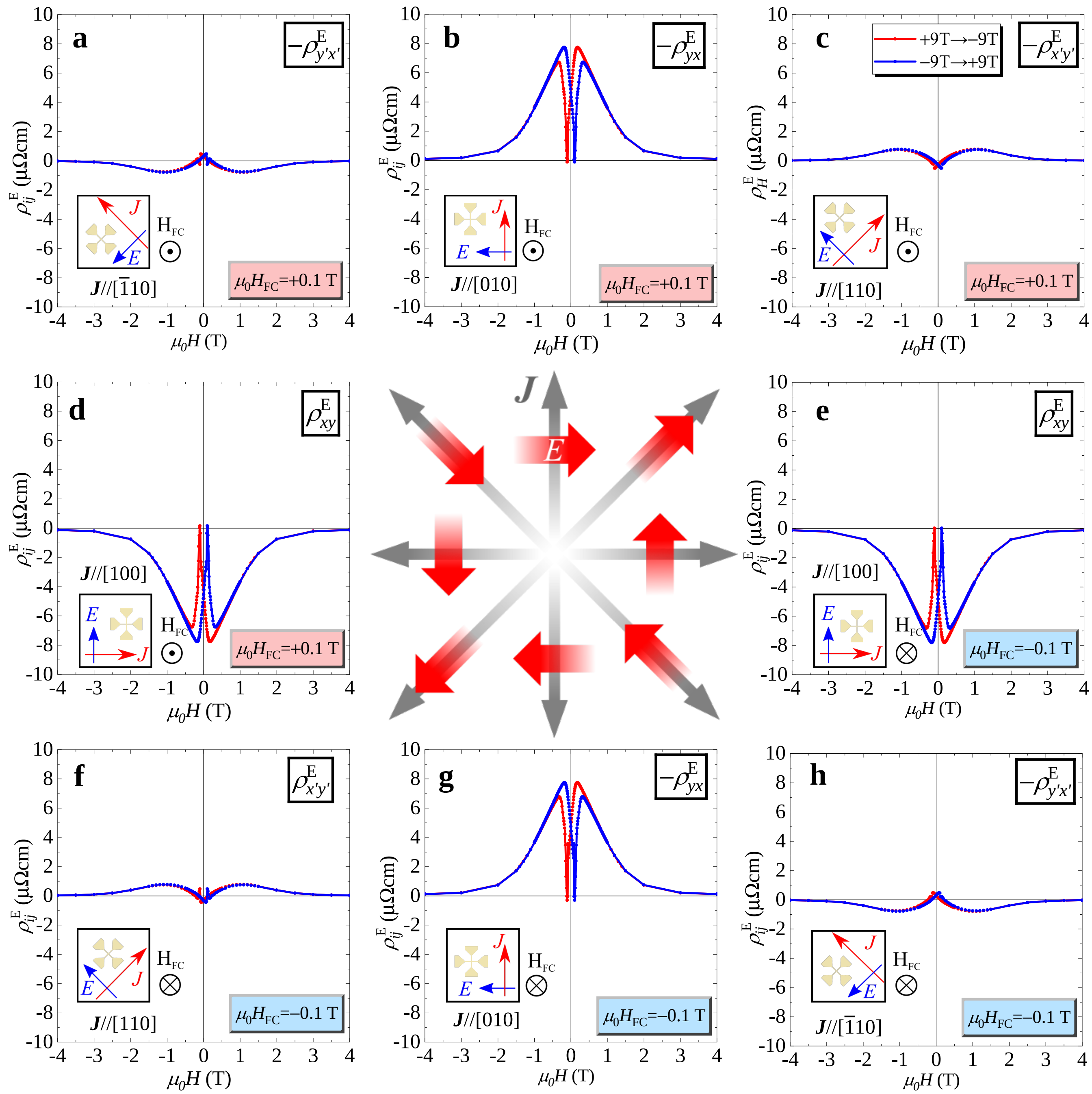

Supplementary Fig. 2. Magnetic field dependence of symmetric transverse resistivity (TR) ρ_{ij}^{E} at 5 K with changing current direction and cooling field. Currents are applied parallel to (**a**, **h**) $\mathbf{J}||[\bar{1}10]$, (**b**, **g**) $\mathbf{J}||[010]$, (**c**, **f**) $\mathbf{J}||[110]$, and (**d**, **e**) $\mathbf{J}||[100]$ axes. TR was measured after field cooling at (**a-d**) $\mu_0 H_{\mathrm{FC}} = +0.1$ T and (**e-h**) $\mu_0 H_{\mathrm{FC}} = -0.1$ T.

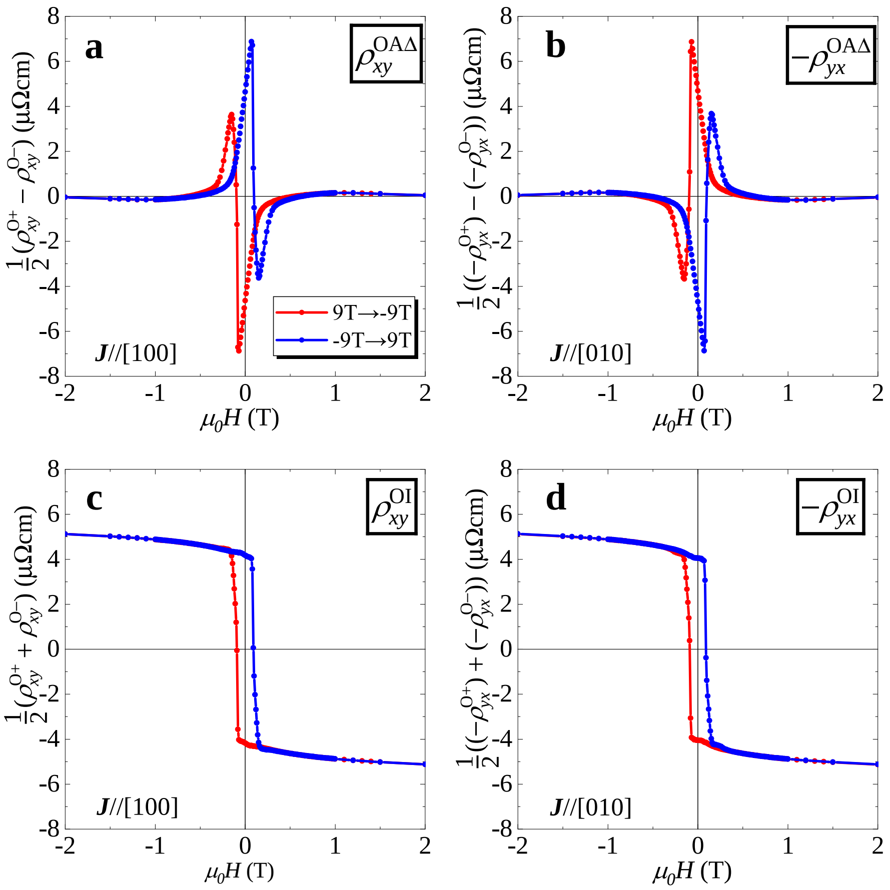

Supplementary Fig. 3. Magnetic field dependence of (**a,b**) difference $[\rho_{ij}^{\mathrm{OA}\Delta} = (\rho_{ij}^{\mathrm{O}+} - \rho_{ij}^{\mathrm{O}-})/2]$ and (**c,d**) average $[\rho_{ij}^{\mathrm{OI}} = (\rho_{ij}^{\mathrm{O}+} + \rho_{ij}^{\mathrm{O}-})/2]$ of the antisymmetric TR between field cooling with $\mu_0 H_{\mathrm{FC}} = +0.1$ T $[\rho_{ij}^{\mathrm{O}+}]$ and $\mu_0 H_{\mathrm{FC}} = -0.1$ T $[\rho_{ij}^{\mathrm{O}-}]$. Currents are applied along (**a,c**) $\mathbf{J}||[100]$ and (**b,d**) $\mathbf{J}||[010]$.

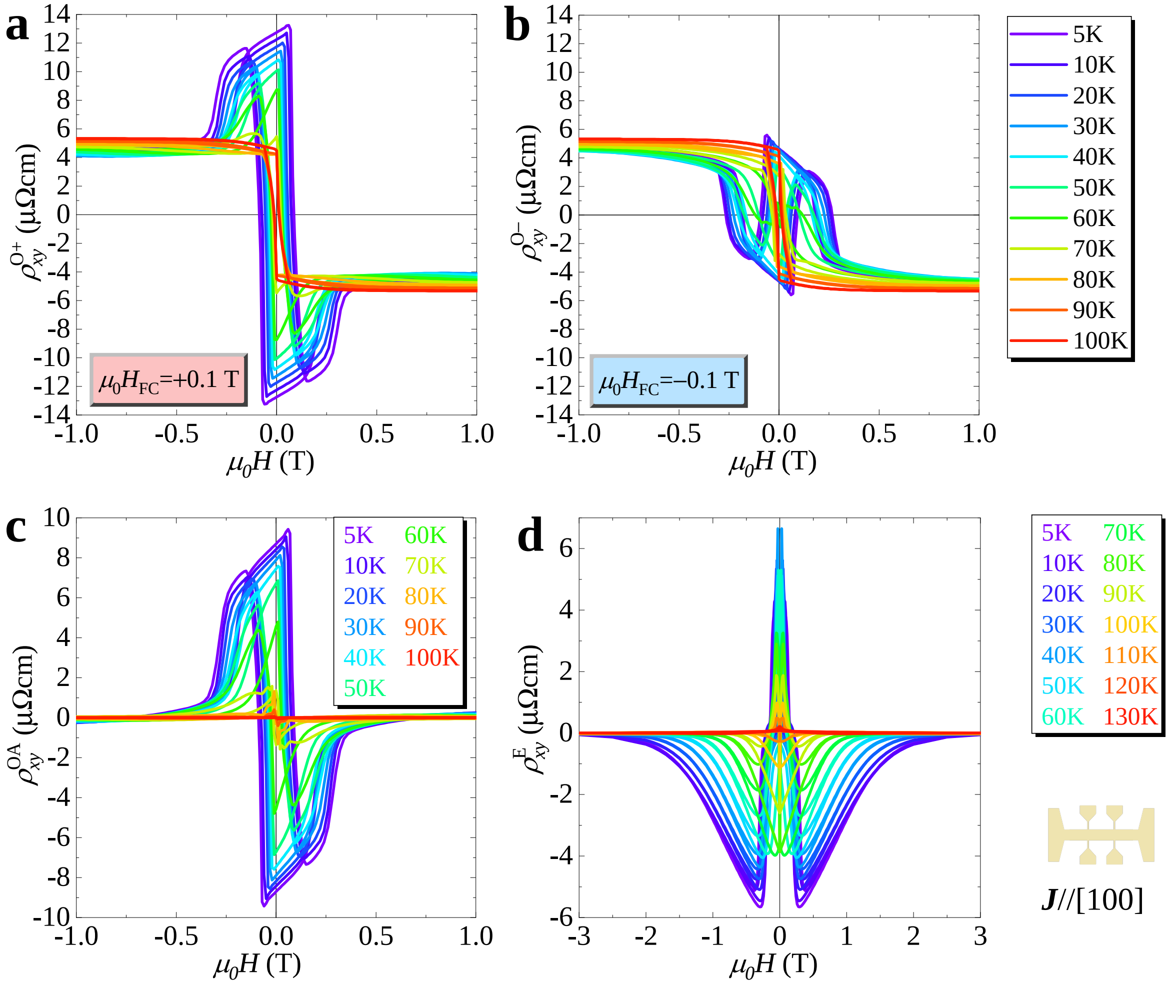

Supplementary Fig. 4. Magnetic field dependence of antisymmetric TR ρ^{O}_{ij} for $\mathbf{J}||[100]$ from 5 K to 100 K measured after field cooling of (**a**) $H_{\mathrm{FC}} = 0.1$ T, (**b**) $H_{\mathrm{FC}} = -0.1$ T, and (**c**) anisotropic resistance ρ^{O}_{ij} extracted from these two data sets. (**d**) Magnetic field dependence of symmetric TR ρ^{E}_{ij} for $\mathbf{J}||[100]$ from 5 K to 130 K.

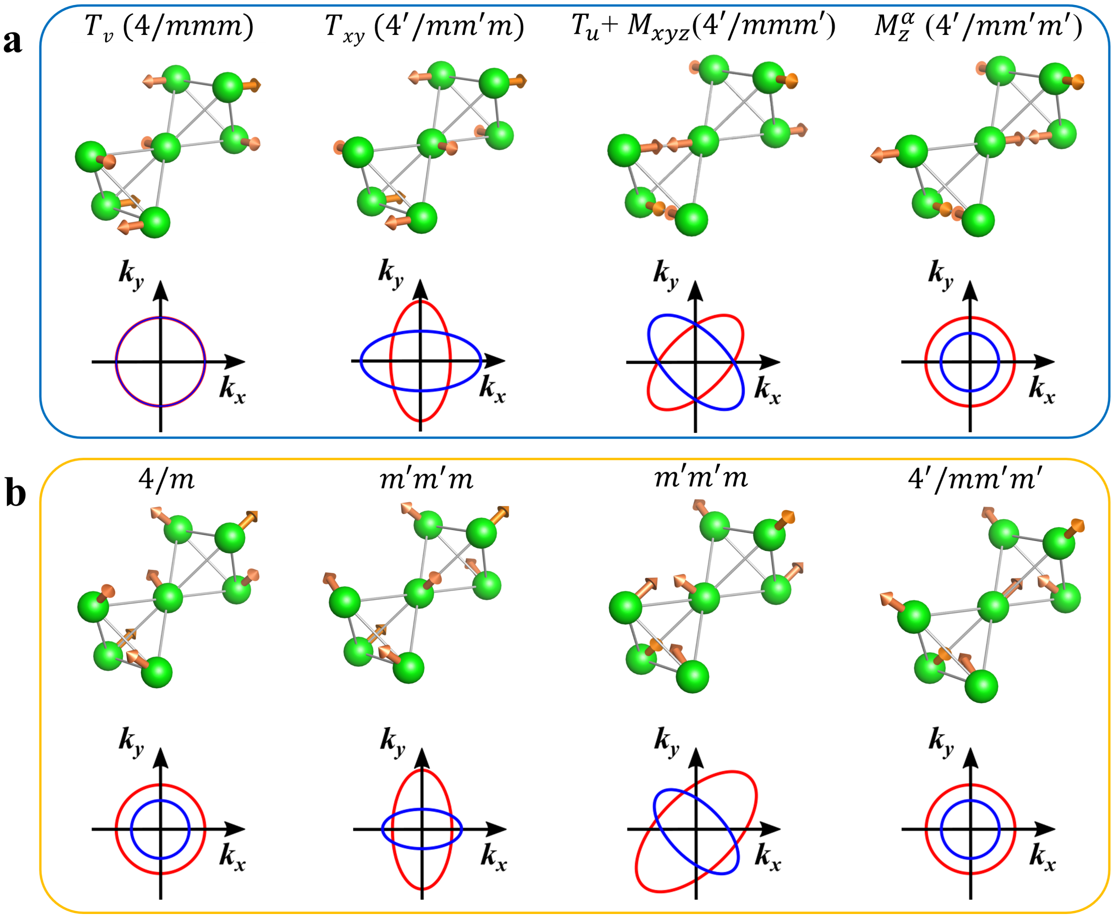

Supplementary Fig. 5. Correspondence between extended magnetic multipoles and band structure. Magnetic structures are considered on pyrochlore lattice for (**a**) in-plane antiferromagnetic structures and (**b**) corresponding conical magnetic structures with net magnetization along the z-direction. The electronic band is depicted in the k_x-k_y plane at $k_z = 0$ with the single band picture (*14*). The red (blue) curves show the up (down)-spin moments along the z-direction.

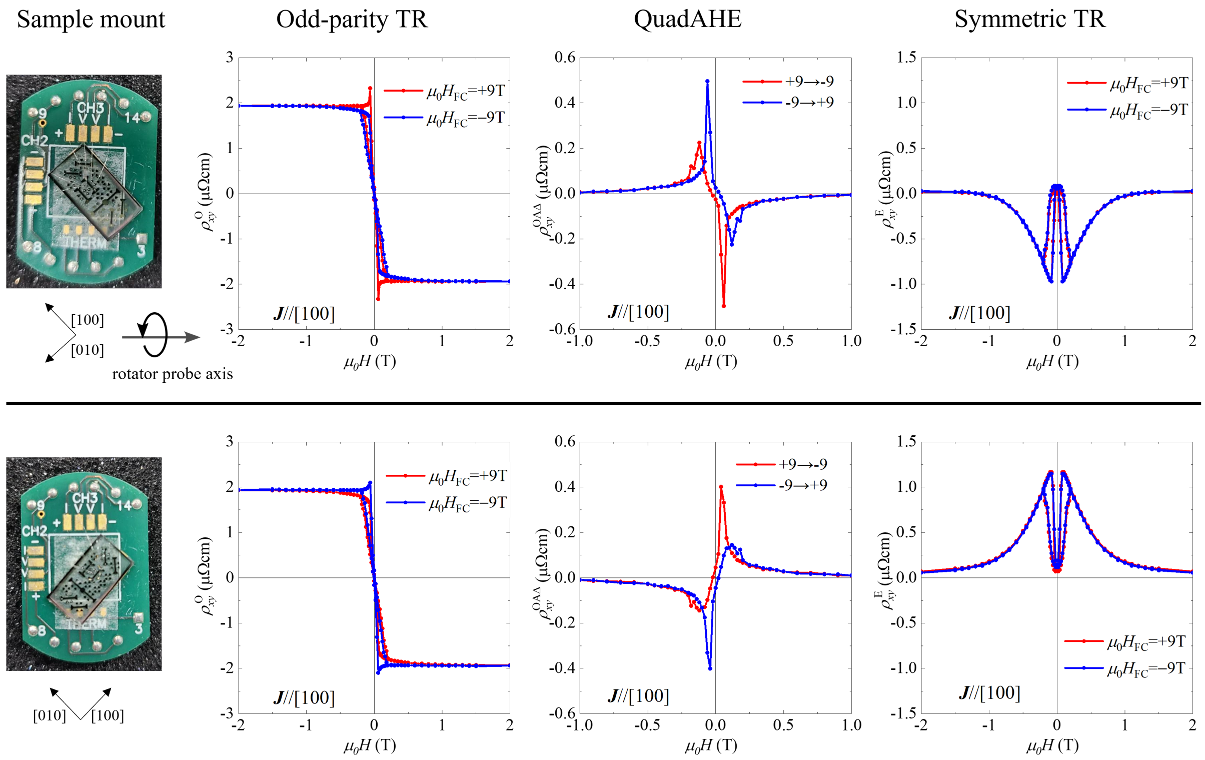

Supplementary Fig. 6. Dependence of transverse resistivity on the sample mounting direction. When the sample was mounted by rotating 90°, the sign of both QuadAHE and symmetric TR reversed, even though the wiring of the electrodes and the direction of the magnetic field applied during cooling remained the same. This result indicates that the change in sign is caused by the in-plane magnetic field resulting from the misalignment of the sample holder.

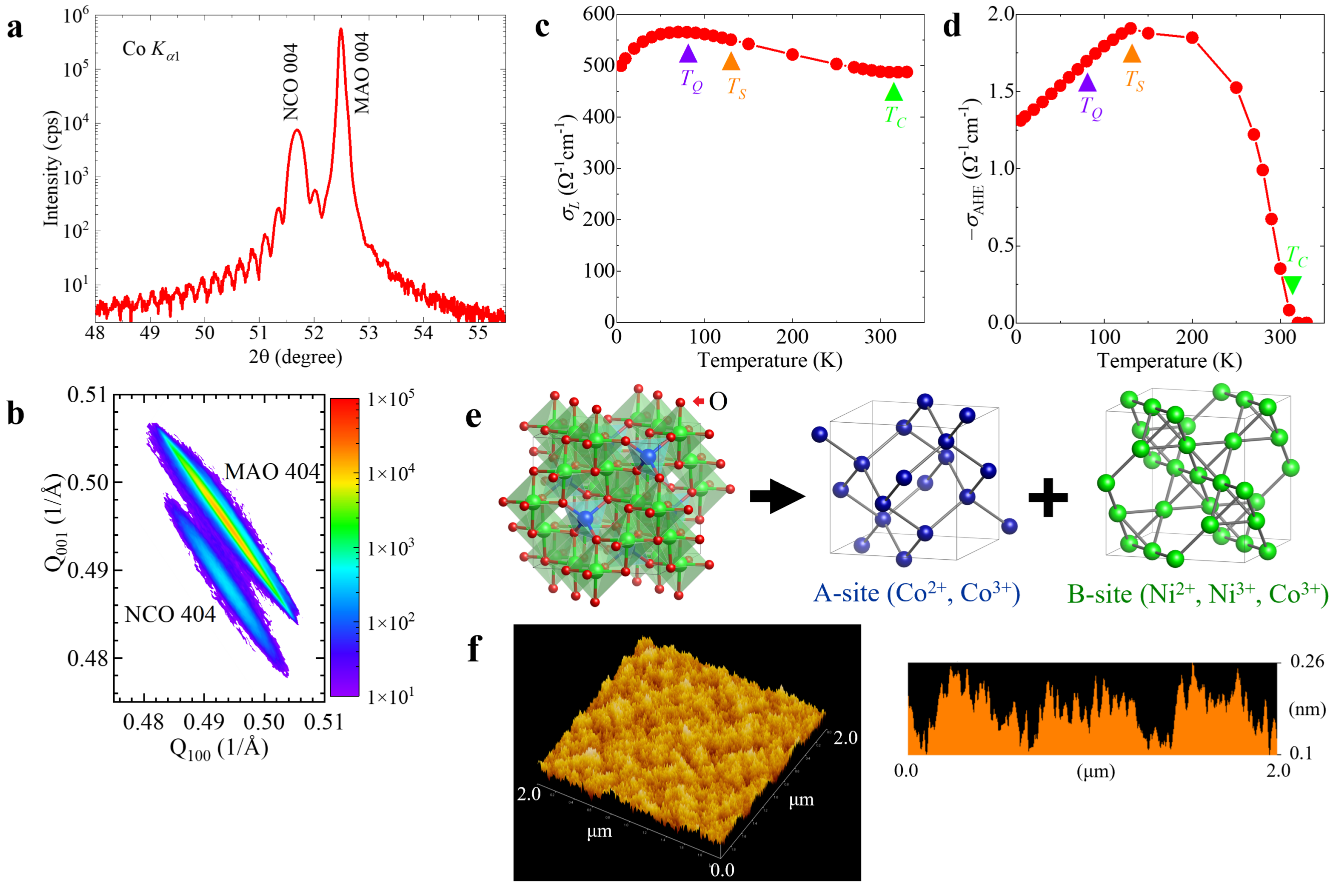

Supplementary Fig. 7. (**a**) X-ray diffraction patterns of $NiCo_2O_4$(NCO)/$MgAl_2O_4$(MAO) thin film for the θ-2θ scan along the (001) direction. (**b**) Reciprocal space contour map of diffraction intensity around 404 Bragg reflections. (**c**) Longitudinal conductivity σ_L (σ_{xx}) and (**d**) Temperature dependence of conventional AHE conductivity σ_{AHE} (σ_{yx}). T_C, T_S, and T_Q indicate magnetic ordering temperatures of the collinear, the Yaffet-Kittle type, and the MTQ conical structures, respectively. (**e**) Crystal structure of spinel oxide with a diamond structure (A-site) and pyrochlore lattice (B-site). (**f**) Atomic force microscopy (AFM) topography image of the thin film.

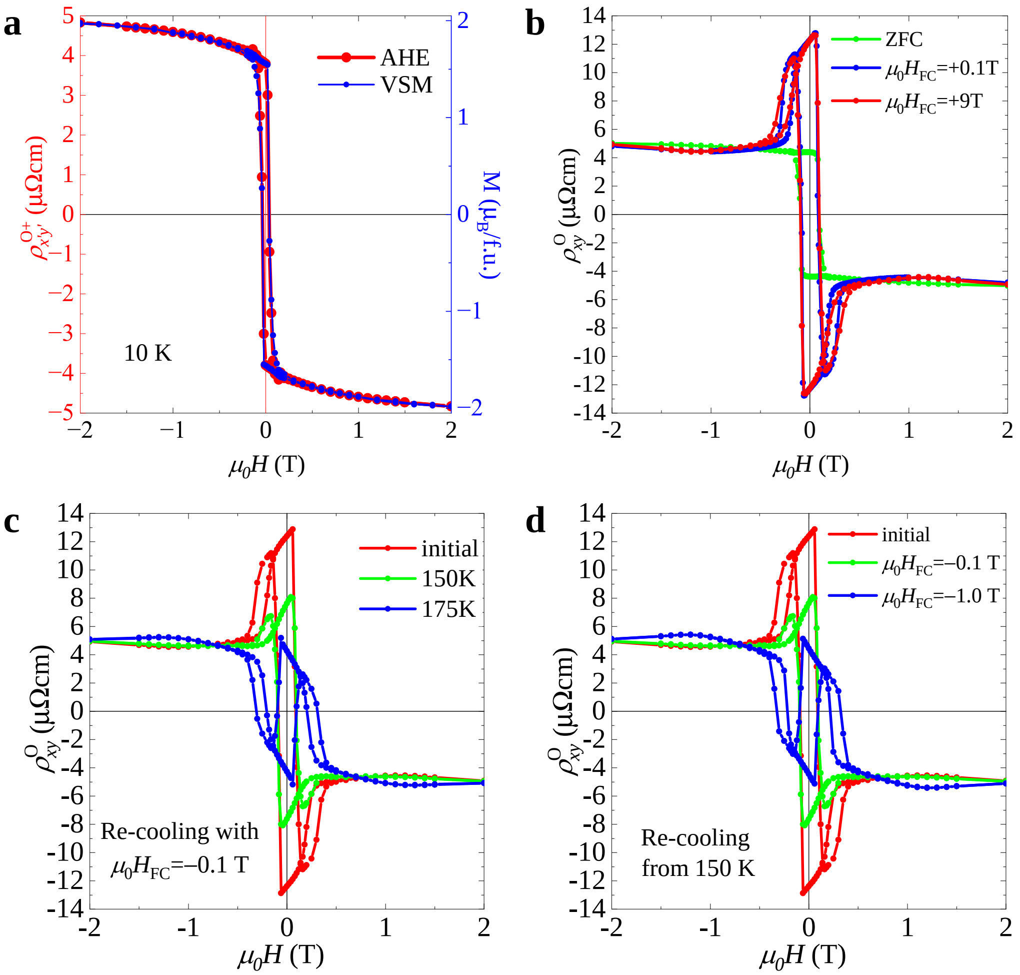

Supplementary Fig. 8. (**a**) Comparison of AHE $\rho^{O\pm}_{x'y'}$ [**J** ∥ [110]] and magnetic hysteresis loop measured by the vibration sample magnetometer (VSM) at 10 K. (**b**) AHE curves dependence on the magnitude of H_{FC}, the zero field cooling (ZFC), 0.1 T and 9 T, in which the cooling was started from 300 K. (**c**) QuadAHE curve dependence on the initial temperature T_s from which the cooling starts. The sample was initially preset by cooling from 300 K to 5 K with $\mu_0 H_{FC} = 0.1$ T. After that, the sample was warmed to specific temperatures, T_s =150 K and 175 K, and then cooled down to 5 K with $\mu_0 H_{FC} = -0.1$ T. (**d**) QuadAHE curve dependence on H_{FC} with T_s = 150 K. The sample was initially preset as well, warmed to 150 K, and then cooled down to 5 K with $\mu_0 H_{FC} = -0.1$ and -1.0 T.

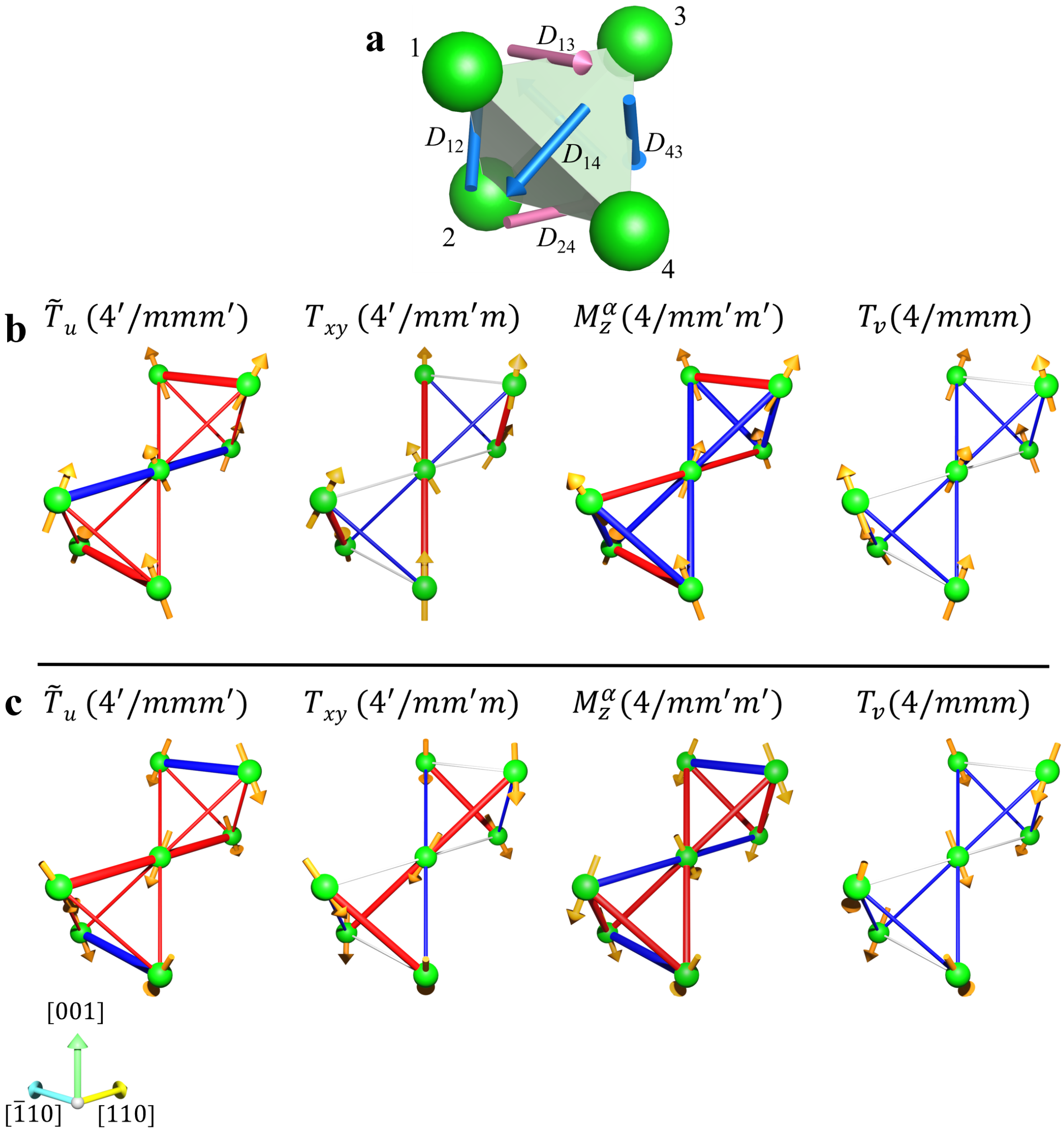

Supplementary Fig. 9. Dzyaloshinskii-Moriya (DM) interaction on pyrochlore lattice. (**a**) DM vectors for each B-B bond on one B-site tetrahedron. The DM interaction energy of each B-B site, $E_{\rm DMI} = \mathbf{D}_{ij} \cdot (\mathbf{S}_i \times \mathbf{S}_j)$, on the possible four conical magnetic structures MTQ $\tilde{T}_u$, T_{xy}, M_z^α, and T_ν for (**b**) $M_z > 0$ and (**c**) $M_z < 0$. The bond radius represents the magnitude of $E_{\rm DMI}$, while red and blue indicate that its sign is positive and negative, respectively. Gray indicates $E_{\rm DMI} = 0$ states.

Supplementary Table I. Centrosymmetric magnetic multipoles ($l = 1, 2, 3$) for the orthonormal magnetic structures on the pyrochlore lattice with the corresponding irreducible representation (IREP), magnetic point group (MPG), and magnetic tensor of charge conductivity and spin conductivity. Only the magnetic multipoles with the principle axis of [001] are adapted from table II in Ref. 16. The charge and spin conductivity tensors are obtained by MTENSOR of Bilbao [17] and Ref. 18.

Magnetic Multipole	IREP	Multipole	MPG	Electric conductivity	Spin conductivity (σ^z)
Magnetic dipole	T_{1g}	M_z	$4/mm'm'$	$\begin{pmatrix} \sigma_{xx} & \sigma_{xy} & 0 \\ -\sigma_{xy} & \sigma_{xx} & 0 \\ 0 & 0 & \sigma_{zz} \end{pmatrix}$	$\begin{pmatrix} \sigma^z_{xx} & \sigma^z_{xy} & 0 \\ -\sigma^z_{xy} & \sigma^z_{xx} & 0 \\ 0 & 0 & \sigma^z_{zz} \end{pmatrix}$
Toroidal quadrupole	E_g	T_v	$4/mmm$	$\begin{pmatrix} \sigma_{xx} & 0 & 0 \\ 0 & \sigma_{xx} & 0 \\ 0 & 0 & \sigma_{zz} \end{pmatrix}$	$\begin{pmatrix} 0 & \sigma^z_{xy} & 0 \\ -\sigma^z_{xy} & 0 & 0 \\ 0 & 0 & 0 \end{pmatrix}$
	E_g	T_u	$4'/mmm'$	$\begin{pmatrix} \sigma_{xx} & 0 & 0 \\ 0 & \sigma_{xx} & 0 \\ 0 & 0 & \sigma_{zz} \end{pmatrix}$	$\begin{pmatrix} 0 & \sigma^z_{xy} & 0 \\ \sigma^z_{yx} & 0 & 0 \\ 0 & 0 & 0 \end{pmatrix}$
	T_{2g}	T_{xy}	$4'/mm'm$	$\begin{pmatrix} \sigma_{xx} & 0 & 0 \\ 0 & \sigma_{xx} & 0 \\ 0 & 0 & \sigma_{zz} \end{pmatrix}$	$\begin{pmatrix} \sigma^z_{xx} & \sigma^z_{xy} & 0 \\ -\sigma^z_{xy} & -\sigma^z_{xx} & 0 \\ 0 & 0 & 0 \end{pmatrix}$
Magnetic octupole	A_{2g}	M_{xyz}	$m\overline{3}m'$	$\begin{pmatrix} \sigma_{xx} & 0 & 0 \\ 0 & \sigma_{xx} & 0 \\ 0 & 0 & \sigma_{xx} \end{pmatrix}$	$\begin{pmatrix} 0 & \sigma^z_{xy} & 0 \\ \sigma^y_{xz} & 0 & 0 \\ 0 & 0 & 0 \end{pmatrix}$
	T_{1g}	M^α_z	$4/mm'm'$	$\begin{pmatrix} \sigma_{xx} & \sigma_{xy} & 0 \\ -\sigma_{xy} & \sigma_{xx} & 0 \\ 0 & 0 & \sigma_{zz} \end{pmatrix}$	$\begin{pmatrix} \sigma^z_{xx} & \sigma^z_{xy} & 0 \\ -\sigma^z_{xy} & \sigma^z_{xx} & 0 \\ 0 & 0 & \sigma^z_{zz} \end{pmatrix}$

[1] Shen, Y., Kan, D., Tan, Z., Wakabayashi, Y. & Shimakawa, Y. Tuning of ferrimagnetism and perpendicular magnetic anisotropy in $NiCo_2O_4$ epitaxial films by the cation distribution. *Physical Review B* **101**, 094412 (2020). URL `https://link.aps.org/doi/10.1103/PhysRevB.101.094412`.

[2] Chen, X. *et al.* Magnetotransport Anomaly in Room-Temperature Ferrimagnetic $NiCo_2O_4$ Thin Films. *Advanced Materials* **31**, 1805260 (2019). URL `https://onlinelibrary.wiley.com/doi/abs/10.1002/adma.201805260`.

[3] Kan, D., Xie, L. & Shimakawa, Y. Scaling of the anomalous Hall effect in perpendicularly magnetized epitaxial films of the ferrimagnet $NiCo_2O_4$. *Physical Review B* **104**, 134407 (2021). URL `https://link.aps.org/doi/10.1103/PhysRevB.104.134407`.

[4] Koizumi, H. *et al.* Spin reorientation in tetragonally distorted spinel oxide $NiCo_2O_4$ epitaxial films. *Physical Review B* **104**, 014422 (2021). URL `https://link.aps.org/doi/10.1103/PhysRevB.104.014422`.

[5] Chen, X. *et al.* Anomalous Hall effect and perpendicular magnetic anisotropy in ultrathin ferrimagnetic NiCo2O4 films. *Applied Physics Letters* **120**, 242401 (2022). URL `https://doi.org/10.1063/5.0097869`. https://pubs.aip.org/aip/apl/article-pdf/doi/10.1063/5.0097869/16447933/242401_1_online.pdf.

[6] Yafet, Y. & Kittel, C. Antiferromagnetic arrangements in ferrites. *Physical Review* **87**, 290–294 (1952). URL `https://link.aps.org/doi/10.1103/PhysRev.87.290`.

[7] Knop, O., Reid, K. I. G., Sutarno & Nakagawa, Y. Chalkogenides of the transition elements. VI. X-Ray, neutron, and magnetic investigation of the spinels Co_3O_4, $NiCo_2O_4$, Co_3S_4, and $NiCo_2S_4$. *Canadian Journal of Chemistry* **46**, 3463–3476 (1968). URL `https://doi.org/10.1139/v68-576`.

[8] Kan, D. *et al.* Spin and orbital magnetic moments in perpendicularly magnetized $Ni_{1-x}Co_{2+y}O_{4-z}$ epitaxial thin films: Effects of site-dependent cation valence states. *Physical Review B* **101**, 224434 (2020). URL `https://link.aps.org/doi/10.1103/PhysRevB.101.224434`.

[9] Hayami, S., Yatsushiro, M., Yanagi, Y. & Kusunose, H. Classification of atomic-scale multipoles under crystallographic point groups and application to linear response tensors. *Physi-*

cal Review B **98**, 165110 (2018). URL https://link.aps.org/doi/10.1103/PhysRevB.98.165110.

[10] Ziman, J. The electron transport properties of pure liquid metals. *Advances in Physics* **16**, 551–580 (1967). URL https://doi.org/10.1080/00018736700101665. https://doi.org/10.1080/00018736700101665.

[11] Nagaosa, N., Sinova, J., Onoda, S., MacDonald, A. H. & Ong, N. P. Anomalous hall effect. *Reviews of Modern Physics* **82**, 1539–1592 (2010). URL https://link.aps.org/doi/10.1103/RevModPhys.82.1539.

[12] Moriya, T. Anisotropic superexchange interaction and weak ferromagnetism. *Physical Review* **120**, 91–98 (1960). URL https://link.aps.org/doi/10.1103/PhysRev.120.91.

[13] Elhajal, M., Canals, B., Sunyer, R. & Lacroix, C. Ordering in the pyrochlore antiferromagnet due to dzyaloshinsky-moriya interactions. *Physical Review B* **71**, 094420 (2005). URL https://link.aps.org/doi/10.1103/PhysRevB.71.094420.

[14] Kotov, V. N., Elhajal, M., Zhitomirsky, M. E. & Mila, F. Dzyaloshinsky-moriya-induced order in the spin-liquid phase of the $s = 1/2$ pyrochlore antiferromagnet. *Physical Review B* **72**, 014421 (2005). URL https://link.aps.org/doi/10.1103/PhysRevB.72.014421.

[15] Ideue, T. *et al.* Effect of lattice geometry on magnon hall effect in ferromagnetic insulators. *Physical Review B* **85**, 134411 (2012). URL https://link.aps.org/doi/10.1103/PhysRevB.85.134411.

[16] Suzuki, M.-T. *et al.* Multipole expansion for magnetic structures: A generation scheme for a symmetry-adapted orthonormal basis set in the crystallographic point group. *Physical Review B* **99**, 174407 (2019). URL https://link.aps.org/doi/10.1103/PhysRevB.99.174407.

[17] Gallego, S. V., Etxebarria, J., Elcoro, L., Tasci, E. S. & Perez-Mato, J. M. Automatic calculation of symmetry-adapted tensors in magnetic and non-magnetic materials: a new tool of the Bilbao Crystallographic Server. *Acta Crystallographica Section A* **75**, 438–447 (2019). URL https://doi.org/10.1107/S2053273319001748.

[18] Seemann, M., Ködderitzsch, D., Wimmer, S. & Ebert, H. Symmetry-imposed shape of linear response tensors. *Physical Review B* **92**, 155138 (2015). URL https://link.aps.org/doi/10.1103/PhysRevB.92.155138.
